# Improving the Detection of Individuals at Clinical Risk for Psychosis in the Community, Primary and Secondary Care: An Integrated Evidence-Based Approach

**DOI:** 10.3389/fpsyt.2019.00774

**Published:** 2019-10-24

**Authors:** Paolo Fusar-Poli, Sarah A. Sullivan, Jai L. Shah, Peter J. Uhlhaas

**Affiliations:** ^1^Early Psychosis: Interventions and Clinical-detection (EPIC) Lab, Department of Psychosis Studies, Institute of Psychiatry, Psychology & Neuroscience, King’s College London, London, United Kingdom; ^2^OASIS service, South London and Maudsley NHS Foundation Trust, London, United Kingdom; ^3^Department of Brain and Behavioral Sciences, University of Pavia, Pavia, Italy; ^4^National Institute for Health Research Maudsley Biomedical Research Centre, South London and Maudsley NHS Foundation Trust, London, United Kingdom; ^5^Centre for Academic Mental Health, Bristol Medical School, University of Bristol, Bristol, United Kingdom; ^6^Prevention and Early Intervention Program for Psychosis (PEPP-Montréal), Douglas Mental Health University Institute, Montréal, QC, Canada; ^7^ACCESS Open Minds (Pan-Canadian Youth Mental Health Services Research Network), Douglas Mental Health University Institute, Montreal, QC, Canada; ^8^Department of Psychiatry, McGill University, Montreal, QC, Canada; ^9^Institute of Neuroscience and Psychology, University of Glasgow, Glasgow, United Kingdom; ^10^Department of Child and Adolescent Psychiatry, Charité Universitätsmedizin, Berlin, Germany

**Keywords:** Clinical high risk, detection, e-health, prevention, psychosis, risk, schizophrenia

## Abstract

**Background:** The first rate-limiting step for improving outcomes of psychosis through preventive interventions in people at clinical high risk for psychosis (CHR-P) is the ability to accurately detect individuals who are at risk for the development of this disorder. Currently, this detection power is sub-optimal.

**Methods:** This is a conceptual and nonsystematic review of the literature, focusing on the work conducted by leading research teams in the field. The results will be structured in the following sections: understanding the CHR-P assessment, validity of the CHR-P as a universal risk state for psychosis, and improving the detection of at-risk individuals in secondary mental health care, in primary care, and in the community.

**Results:** CHR-P instruments can provide adequate prognostic accuracy for the prediction of psychosis provided that they are employed in samples who have undergone risk enrichment during recruitment. This substantially limits their detection power in real-world settings. Furthermore, there is initial evidence that not all cases of psychosis onset are preceded by a CHR-P stage. A transdiagnostic individualized risk calculator could be used to automatically screen secondary mental health care medical notes to detect those at risk of psychosis and refer them to standard CHR-P assessment. Similar risk estimation tools for use in primary care are under development and promise to boost the detection of patients at risk in this setting. To improve the detection of young people who may be at risk of psychosis in the community, it is necessary to adopt digital and/or sequential screening approaches. These solutions are based on recent scientific evidence and have potential for implementation internationally.

**Conclusions:** The best strategy to improve the detection of patients at risk for psychosis is to implement a clinical research program that integrates different but complementary detection approaches across community, primary, and secondary care. These solutions are based on recent scientific advancements in the development of risk estimation tools and e-health approaches and have the potential to be applied across different clinical settings.

## Introduction

**Figure 1:**
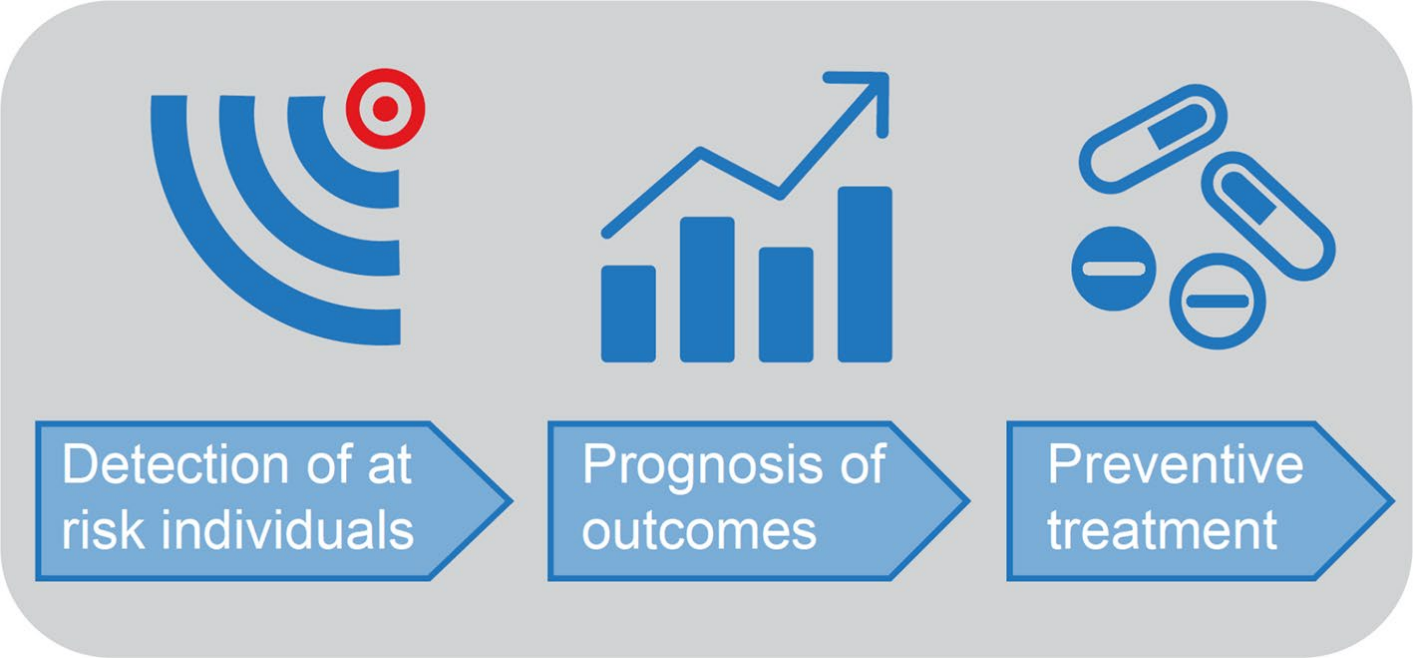
Core clinical components for effective prevention of psychosis. The first rate-limiting step for improving outcomes of psychosis through preventive approaches is the ability to accurately detect individuals at risk for psychosis. Adapted from (14), Creative Commons Attribution License (CC BY).

Preventive strategies in young people at clinical high risk for psychosis [CHR-P (1)] can ameliorate the high personal, familial, societal, and clinical burden of psychotic disorders (2). CHR-P criteria, which include the ultra-high-risk state [e.g., at-risk mental state (3) or other psychosis-risk syndromes (4)] and/or basic symptoms (5), are detected by specialized clinical services (6) through established psychometric assessment tools (7), in the context of a clinical interview (8). These tools are internationally validated (7) and assess whether the individual is meeting at least one of the three ultra-high-risk subgroups: attenuated psychotic symptoms (∼85% of cases), genetic risk and deterioration syndrome (5% of cases), or brief and limited intermittent psychotic symptoms (BLIPS, 10% of cases) (3, 9) subgroup. Individuals at CHR-P recruited from help-seeking clinical samples have a 20% probability of developing emerging psychotic disorders (but not other nonpsychotic disorders (10, 11)) over 2 years (12). This risk increases to 50% at 2 years for the BLIPS subgroup and to 89% at 5 years for the subset of BLIPS patients who present with seriously disorganizing and dangerous features (13). Overall, the real-world potential impact of the CHR-P paradigm for improving the outcomes of psychotic disorders will be determined by the successful and stepped integration of three key components (**Figure 1**): (i) efficient detection of individuals at risk for psychosis, (ii) accurate prognosis of outcomes, and (iii) effective preventive treatment.

**Figure 2:**
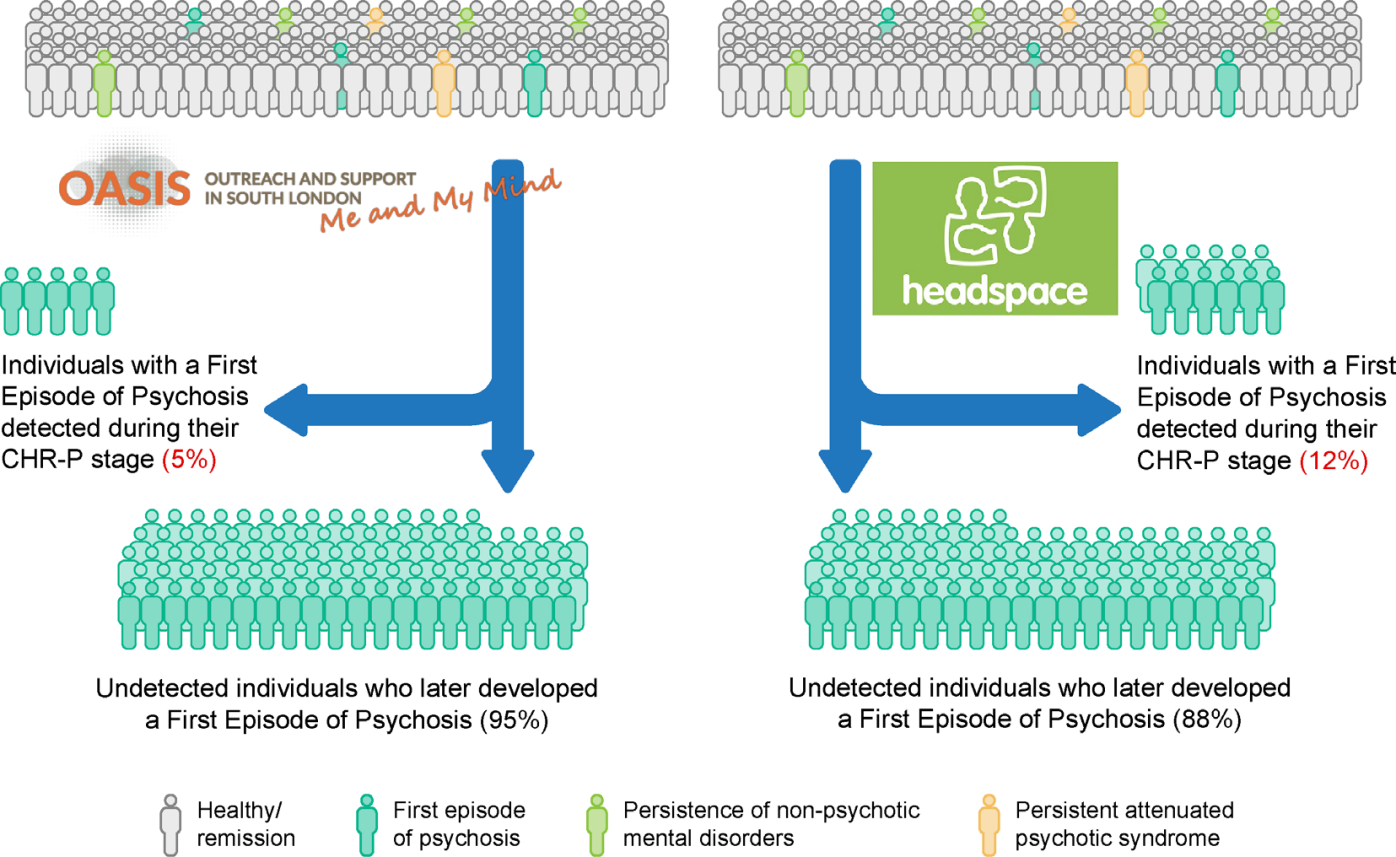
Detection power of at-risk patients who will later develop a first-episode of psychosis under different preventive programs: OASIS and headspace. CHR-P: Clinical High Risk for Psychosis. New figure.

As illustrated in **Figure 1**, the first rate-limiting step for improving outcomes of psychosis through the CHR-P paradigm is the real-world ability to detect most individuals who are at risk for psychosis and will later develop it. Efficient detection of individuals at CHR-P has been a relatively neglected area of research in spite of the fact that inefficient detection impedes subsequent efforts. In fact, even the most accurate prognostic model and effective preventive treatment would exert a modest impact if they are only applied to a small proportion of those who later develop psychosis. The first challenge is that, to date, there has been an assumption that the CHR-P stage represents the prototypical prepsychotic stage for most individuals who will later go on to develop psychosis. However, in a thematic issue in Schizophrenia Bulletin titled “Dissecting the diagnostic pluripotentiality of the ultra high risk state for psychosis,” (Volume 44, Issue 2, 2018) (15–18), a meta-analysis demonstrated that the onset of psychosis may also occur via previously identified nonpsychotic clinical risk syndromes (17). Separately, independent research groups have reported that first-episode psychosis (FEP) cases may occur without a prior identifiable period of subthreshold psychotic symptoms (19, 20). The second challenge is that even assuming that the CHR-P concept would be sufficient to detect the majority of individuals at risk, its real-world penetrance is undetermined. Emerging evidence suggests that current detection strategies for identifying individuals at CHR-P are highly inefficient. These strategies are largely based on referrals to specialized CHR-P clinics (6), made on suspicion of psychosis risk. Only 5% of individuals who had presented with a first onset of nonorganic psychosis to the local NHS Trust had been detected by one local CHR-P service (21). Since the service had been fully established in the same Trust, there is a clear need to improve the detection of at-risk cases (22). To our best knowledge, there are no other original studies published to date reporting on the detection power of the CHR-P paradigm that could further validate or replicate these findings. Inefficient detection has important clinical implications. For example, although the NHS England’s Access and Waiting Times-Standard for Early Intervention in psychosis (23) requires that CHR-P are detected nationwide and treated within 2 weeks, current detection strategies are inefficient. A first viable alternative may be to intensify the outreach campaigns currently adopted by CHR-P clinics. Converging evidence has demonstrated that such an approach conflicts with the intrinsic psychometric limitations of the CHR-P interviews, producing a diluted transition risk (24, 25) and unreliable prognostic accuracy. Another option may be to implement front-line youth mental health services such as the Headspace initiative (other youth mental health services are available worldwide; for a recent review, see (26)). Because of their one-stop-shop nature (26–28), youth-friendly services are expected to improve the attraction and detection of potential individuals who may be at risk of psychosis. Unfortunately, there are no original data reporting on the efficacy of detecting individuals at CHR-P through youth mental health services. Rough estimates indicate only a modest improvement of detection when adopting broad youth mental health services, with 12% of individuals with FEP being detected at the time of their CHR-P phase (29) (**Figure 2**). Therefore, at present, between 88% (Headspace model) and 95% [Outreach and Support in South London (OASIS) model] of individuals who will later develop psychosis remain undetected at the time of their CHR-P stage (see **Figure 2**).

In order to extend the preventive benefits of the CHR-P paradigm, more sophisticated and innovative approaches are urgently needed (30).

The current manuscript will review this issue in a comprehensive conceptual analysis of the current challenges and propose evidence-based ways for overcoming them. The detection program presented here integrates three separate approaches targeting different populations: secondary mental health care, primary care, and the community. The overarching methodology of this detection program leverages the recent advancements brought by clinical risk estimation tools (31) and digital approaches.

## Method

This is a conceptual but nonsystematic review of the literature, which focuses on the areas of work conducted by our research teams. As such, the information included here largely reflects our conceptual opinion regarding the best path forward an improved detection of CHR-P individuals. We will first review the conceptual foundation of the CHR-P assessments, a necessary step to grasp their intrinsic limitations. Following this analysis, we will appraise the conceptual validity of the CHR-P stage as a universal and prototypical risk state for psychosis. Then, we will propose empirical ways for improving the detection of CHR-P individuals. The results are structured in the following sections: understanding the CHR-P assessment, validity of the CHR-P as universal risk state for psychosis, improving the detection of at-risk individuals in secondary mental health care, improving the detection of psychosis in primary care, and improving the detection of psychosis in the community.

## Results

### Understanding The CHR-P Assessment

**Figure 3:**
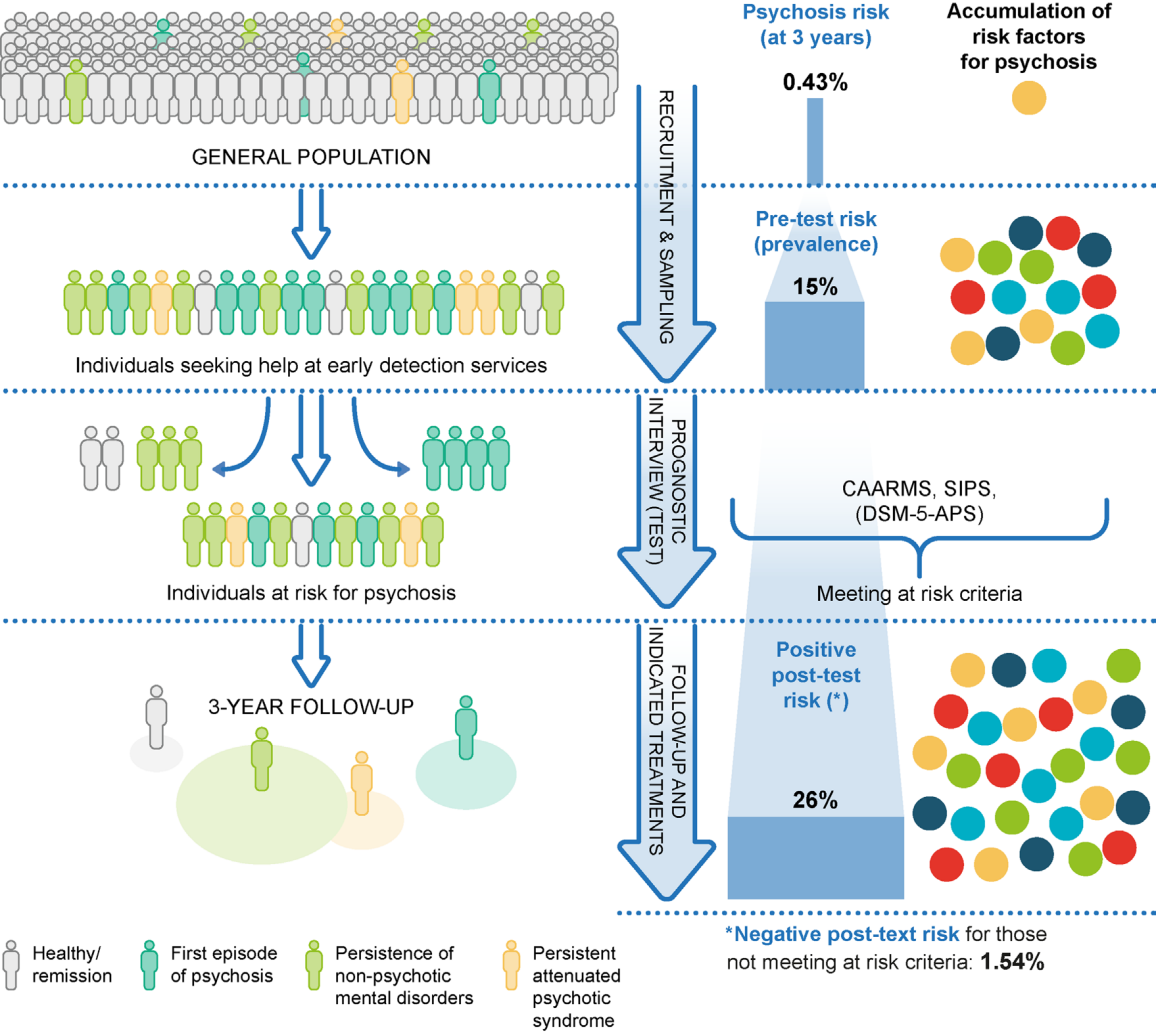
Sampling procedure for individuals at clinical high risk for psychosis (CHR-P) (33). Idiosyncratic recruitment strategies that are characterized by heterogeneous sampling biases (convenience and judgmental sampling) result in accumulation of various risk factors for psychosis and differential level of enrichment of psychosis risk. The risk before the CHR-P assessment is completed is termed pretest risk or prevalence. The risk after the CHR-P assessment is completed is termed posttest risk (positive if CHR-P criteria are met and negative if CHR-P criteria are not met). The figure is based on the data reported in (32, 34, 35). CAARMS: Comprehensive Assessment of At-Risk Mental States; SIPS: Structured Interviews for Psychosis-Risk Syndromes; DSM-5-APS: Diagnostic and Statistical Manual, 5th Edition, Attenuated Psychosis Syndrome. Adapted from (36), Creative Commons Attribution License (CC BY).

CHR-P cohorts are not representative of the local general population because recruitment is affected by sampling biases. To exemplify this, in the general population of South London, the cumulative 3-year incidence of psychotic disorders is 0.43% (32) (**Figure 3**).

**Figure 4:**
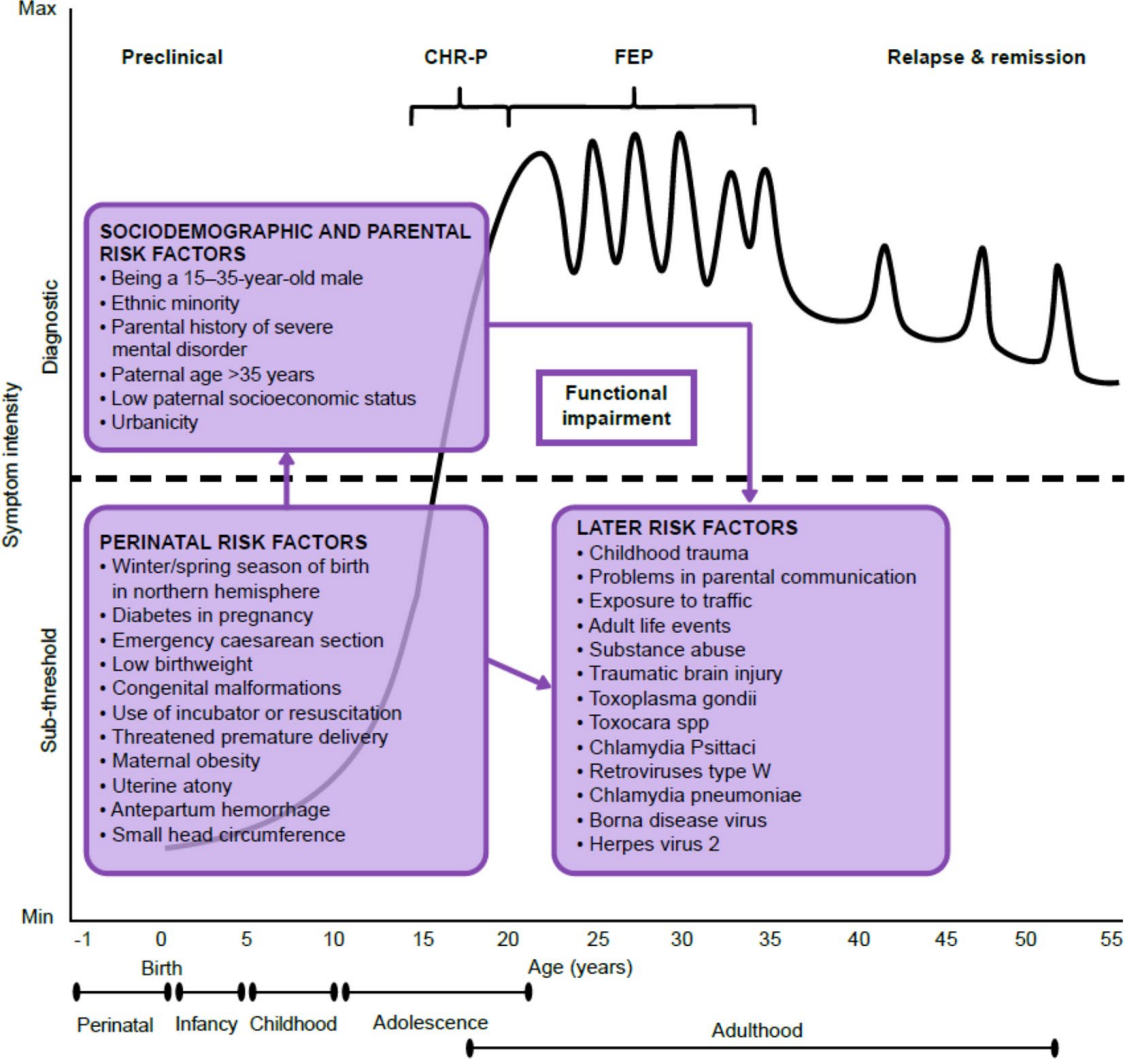
Putative model of the onset and progression of psychosis in relation to nonpurely genetic risk factors and developmental processes affected by the disorder. Sociodemographic and parental and perinatal risk factors have been implicated during the preclinical phase, usually observed from the birth to infancy, childhood, and early adolescence. Additional later factors occurring during later adolescence and early adulthood can trigger the onset of attenuated psychotic symptoms, functional impairment, and help-seeking behavior, which constitute the CHR-P stage. The diagnosis of psychosis, which operationally corresponds to FEP, is usually made during the adolescence or early adulthood, with a peak at 15–35 years of age (38). Once diagnosed, psychosis usually follows a fluctuating course punctuated by acute exacerbation of psychotic crises superimposed upon a background of poorly controlled negative, neurocognitive, and social cognitive symptoms. The pink boxes represent the risk factors for psychosis as identified by a recent umbrella review (38). There is no assumption that these risk factors are of causal nature or that they are independent from each other. Furthermore, certain risk factors may actually represent outcomes of earlier risk factors. Figure based on the data reported in (22). CHR-P: clinical high risk for psychosis; FEP: first-episode psychosis. Adapted from (36), Creative Commons Attribution License (CC BY).

The recruitment of individuals for undergoing a CHR-P assessment is primarily based on unstructured and heterogeneous selection and sampling strategies based on the clinicians’ suspicion of psychosis risk (33) and help-seeking behavior (37). These recruitment processes determine the extent to which individuals at CHR-P would accumulate several risk factors for psychosis (**Figure 3**) (22, 38); in turn, the accumulation of risk factors determines the level of functional impairment (39, 40) and associated attenuated psychotic symptoms (**Figure 4**) (8). Broadly speaking, individuals are generally recruited from secondary mental health care, primary care, or the community and represent different populations on the basis of clinical and functional characteristics. The current manuscript will be structured around strategies to detect these three different populations.

The type of recruitment strategies adopted will influence the level of risk of psychosis for these individuals. This level of risk is also defined as pretest risk (or prevalence) because it is ascertained in the whole group of people undergoing a CHR-P assessment before the results of the assessment itself are known (41). The relative increase in enrichment in this pretest risk, which is acquired through the recruitment step, is substantial (i.e., from 0.43 to 15%, ∼35-fold higher). This pretest risk enrichment is also highly heterogeneous across different sites because it is unstandardized and not controlled for (32, 33, 42). For example, it is highest if recruitment targets secondary mental health care, intermediate if recruitment targets primary care, and lowest if it targets the nonhelp-seeking community (33). Clinical help-seeking samples who undergo pretest risk enrichment during the recruitment phase are then tested by specialized clinics (6). These clinics administer a comprehensive psychometric CHR-P assessment in the context of a clinical interview (43). Overall, a meta-analysis has confirmed that the prognostic accuracy of this CHR-P assessment is considered to be good (i.e., area under the curve at 38 months = 0.90, 95%CI 0.87–0.93) (7) and comparable to that of similar prognostic measurements employed in other areas of medicine (7). As illustrated in **Figure 3**, when help-seeking individuals presenting to a CHR-P service with a 15% pretest risk at 3 years are assessed (tested), those who meet CHR-P criteria will have a 26% risk of developing psychosis at 3 years (1.7-fold increase) and those who do not meet the CHR-P criteria will have a 1.56% risk of developing psychosis at 3 years (10-fold decrease). However, these numbers indicate that the CHR-P tools can accurately predict the onset of psychosis (but not of other nonpsychotic mental disorders (11)) in samples who have been enriched in their risk for developing psychosis (7). If these tools are used to screen the general population, the pretest risk would be low, and even meeting CHR-P criteria would be associated with only a 5% risk of developing psychosis at ∼3 years (24, 25). In other words, the overall accuracy of the CHR-P assessment is driven by a high power to rule out a state of risk for psychosis in samples that are risk enriched, but only a modest capacity to rule in a state of risk for psychosis (7).

These arguments clearly indicate that the CHR-P paradigm has the greatest utility when used to detect help-seeking populations that are accessing specialized clinical services (6). Intensifying outreach campaigns targeting the community would reduce the pretest risk and, in turn, dilute the prognostic accuracy of the CHR-P approach, thereby impeding effective preventive interventions. These considerations will be used to inform the detection approach proposed in the following sections.

### Validity Of The CHR-P Paradigm As Universal Risk State For Psychosis

Most contemporary research on transitions from an at-risk state to FEP has been conducted with help-seeking individuals who are identified as being in CHR-P states. While this is undoubtedly valuable in its own right, there is emerging evidence that identification and intervention at the point of CHR-P currently detect only a small proportion of patients who eventually develop FEP (21). These findings dovetail with the sampling biases that characterize CHR-P studies (44) and, from a public health perspective, lead to the question of what proportion of FEP cases were in fact preceded by a CHR-P state.

The contemporary meta-analytical literature has revealed that reported risk of conversion from a CHR-P stage to FEP (29% at 2 years in 2012 (45)) has decreased internationally in recent years (20% at 2 years in 2016 (12)). However, this is not universal; for example, in South London, the risk of psychosis has remained stable over two decades (42). There is evidence suggesting that the decline in transition is linked to a change in recruitment strategies (42). Whatever the impact of recruitment strategies on the risk for psychosis onset, there is no evidence that the declining conversion rates in the most recent years have been matched by a similar change in the incidence of FEP (46–48). This implies that FEP cases passing through a CHR-P state are not being identified by existing CHR-P research and clinical infrastructures and/or that some individuals developed FEP without experiencing an identified preonset CHR-P state (19, 20). Congruent with this, it has been speculated that, in community samples, those who develop FEP may vary in their clinical backgrounds and outcomes to a greater extent than in those presenting to academic institutions (49).

**Figure 5:**
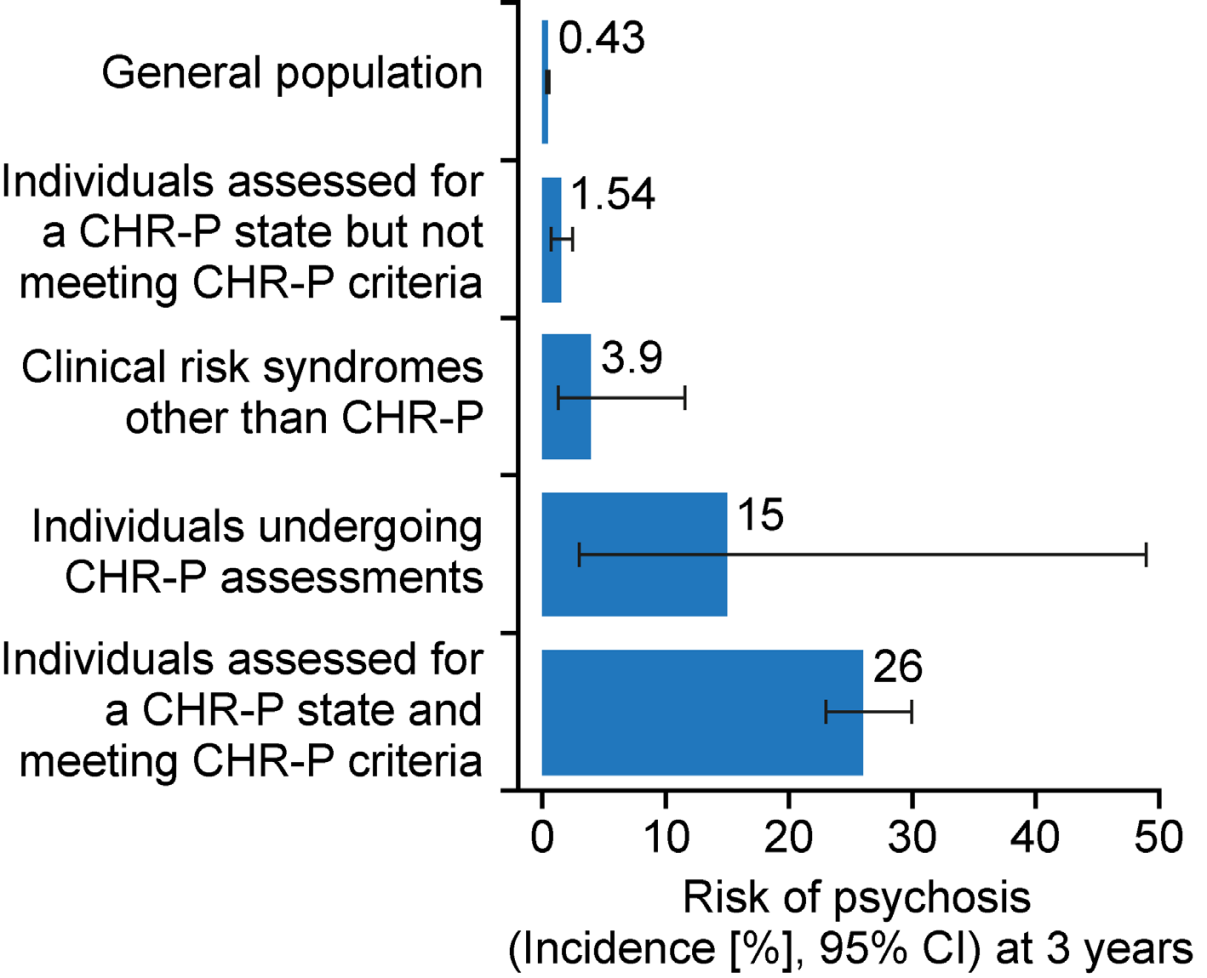
Three-year risk of developing psychosis in different samples at risk. The incidence of psychotic disorders in the general population is significantly influenced by geographical, ethnical, environmental, and the diagnostic criteria of psychosis. However, it can be approximated at 0.43% at 3 years. Help-seeking samples that undergo a CHR-P assessment have a 15% risk of psychosis at 3 years. After the assessment is completed, those who do not meet the CHR-P criteria have a 1.54% risk of psychosis at 3 years, while those who meet the CHR-P criteria have a 26% risk at 3 years. Clinical risk syndromes other than psychosis have a 3.9% risk of psychosis at 3 years. New figure using data from (17, 36). CHR-P: Clinical High Risk for Psychosis.

First, the possibility that nonpsychotic risk syndromes could precede the first onset of psychosis has been demonstrated for some time and was recently summarized in a meta-analysis (17). Within prospective studies (*n* = 4, sample = 1,051), the pooled incidence of new psychotic disorders across these clinical risk syndromes was of 12.9 per 1,000 person-years. Within the same prospective studies, the incidence of common (nonpsychotic) disorders (*n* = 3, sample = 538) was of 43.5 per 1,000 person-years (95% CI: 30.9, 61.3) (17). The study concluded that nonpsychotic risk states may give rise to psychotic disorders, albeit at lower rates than in the CHR-P group (**Figure 5**).

**Figure 6:**
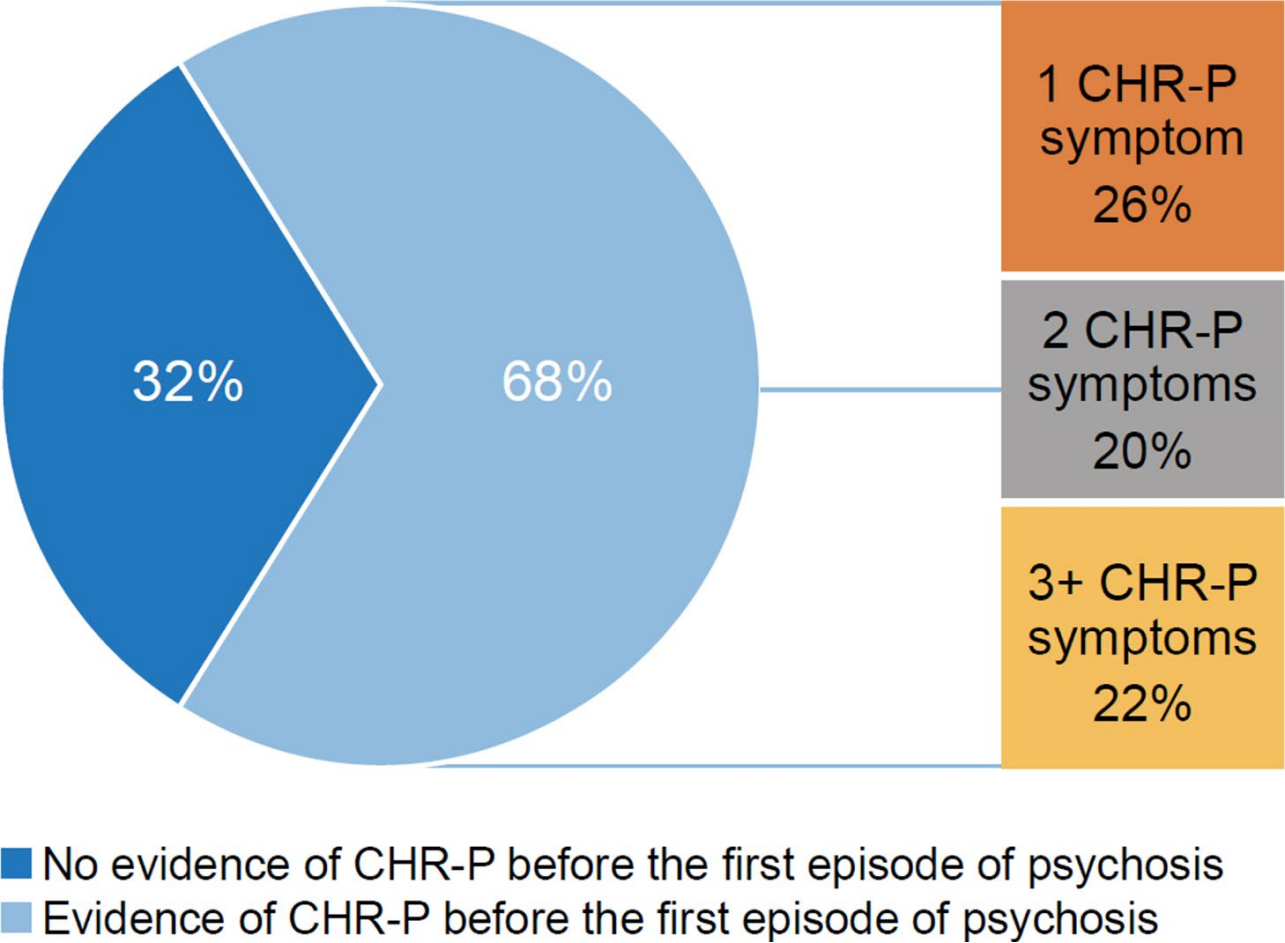
Proportion of patients with first episode psychosis (FEP) who presented with subthreshold psychotic symptoms (consistent with a theoretical CHR-P stage) or not before developing FEP, retrospective analysis of medical records. CHR-P: clinical high risk for psychosis; FEP: first episode psychosis. New figure using data from (19).

Second, although the CHR-P state is not associated with an increased risk of developing new or emerging nonpsychotic mental disorders (10), at follow-up, many of them have other mental illnesses that were already present at baseline, in particular, depressive, anxiety, or substance-use disorders (50, 51). Since individuals at CHR-P often develop nonpsychotic disorders, it is also plausible that some individuals experiencing FEP had developed this without a prior CHR-P syndrome (i.e., without any past presence of subthreshold psychotic symptoms). Indeed, recently two retrospective cohort studies using different instruments each found a reasonably large subgroup of patients with FEP for whom there was no evidence of meeting prior CHR-P criteria for any identifiable length of time (19, 20). This cumulates to ∼30% of the cases experiencing FEP (**Figure 6**).

Subsequent work has explored the longitudinal evolution of patients with FEP who did versus did not experience a preonset CHR-P stage. While there were no clinical or functional differences at baseline (entry to early intervention services) between patients with FEP with and without prior CHR-P states, such differences emerged after 1 year of early intervention services: those with preonset symptoms consistent with a CHR-P state had poorer psychotic symptom outcomes and global functioning (52). Furthermore, there is more frequent nonadherence to antipsychotic medication in the preonset/CHR-P state group (although without corresponding differences in insight) (53). Since this work involved retrospective assessments, it is possible that FEP cases without evidence of a preonset CHR-P phase exhibited a recall bias and that the true prevalence of symptoms consistent with a CHR-P state was substantially higher than measured. Nonetheless, it indicates that the CHR-P stage may not be the unique, universal clinical stage preceding the onset of psychosis. Therefore, to detect more individuals at risk for psychosis, it may be necessary to go beyond the CHR-P operationalization and to adopt a broader transdiagnostic approach (54) that cuts across psychopathological dimensions. For example, there is evidence that a first episode of schizophrenia-like psychosis can occur from depressive or bipolar disorders (22). This concept has informed the development of transdiagnostic risk calculators for this population, as detailed in the following section.

### Improving The Detection Of Individuals At Risk In Secondary Mental Health Care

As noted in the introduction, most individuals accessing the mental health trust in South London who later developed psychosis were not detected at the time of their potential CHR-P stage. This happened in spite of the long-standing implementation of the local specialized CHR-P clinic, the OASIS (6) over the previous two decades, which was conducting an extensive outreach campaign. For example, the clinic uses a youth-friendly website to promote help-seeking behavior and referrals (https://www.meandmymind.nhs.uk). As noted above, it is possible to estimate that up to two-thirds of these FEP cases developed their first onset of the disorder through a CHR-P like stage. As such, the majority of the individuals who developed psychosis would have been detected had these individuals been referred to the local CHR-P (OASIS) clinic. Importantly, all these young people were already under the care of a mental health team. As such, they clearly represent a window of missed opportunities for improving the detection of individuals at risk. Targeting this population would, therefore, be the most obvious first step towards improved detection of at-risk individuals. Within individuals in secondary mental health care, there is an incidence of psychosis of 3% at 6 years, which is higher than the risk of psychosis of 0.62 at 6 years in the local general population (22). The solution to this problem is not simple. One way would be to screen all patients accessing the local mental health trust using the existing CHR-P instruments. This option is logistically and financially unsustainable. The alternative may be to intensify outreach campaigns. However, as noted above (33), these are highly inefficient and dilute the pretest risk of psychosis and, consequently, the prognostic meaningfulness of meeting CHR-P criteria per se.

To overcome this substantial challenge, a clinically based, individualized, transdiagnostic risk calculator has been developed, which includes features that help improve the detection of individuals at risk for psychosis. First, this risk calculator has been externally validated twice: in South London and Maudsley NHS Trust and in Camden and Islington NHS Trust (14, 22, 55). External validation of prognostic models in psychiatry is infrequent (31). Second, this calculator could be applied to mental health trusts where there are no established CHR-P programs to detect patients at risk as in the Camden and Islington Mental Health Trust. Third, this calculator is low cost and simple to run because it uses 10th revision of the International Statistical Classification of Diseases and Related Health Problems index diagnoses (which is considered transdiagnostic because it allows several diagnostic spectra (54)), age, gender, age by gender, and ethnicity as key predictors, which have been selected on the basis of *a priori* clinical knowledge (31, 56). A recent version of the refined calculator that includes an advanced age predictor is also available (57). Fourth, the calculator is deliberately transdiagnostic and includes those meeting the CHR-P state as well as patients who might develop psychosis outside it, meaning that it can potentially detect the subgroup of patients who will go on to develop psychosis outside the CHR-P state. Fifth, the calculator can be automatized because it leverages electronic health records to screen secondary mental health care trusts. Therefore, it has great potential to be applied at scale, which is an essential prerequisite to improve the detection of patients at risk for psychosis. Sixth, the calculator is individualized, in that it provides prognostic outcomes at the individual subject level. This is a substantial advantage compared with the current CHR-P strategy, which is limited by group-level prediction, at risk or not at risk, with few exceptions such as the risk calculator by Cannon et al (58, 59). However, Cannon's risk calculator (58, 59) should be used only in individuals already meeting CHR-P criteria to predict their clinical outcomes; as such, Cannon's algorithm is not suited to improve the detection of individuals at risk in primary, secondary care, or in the community. Seventh, the transdiagnostic calculator can be further improved by the addition of more sophisticated predictors or by the stepped combination of sequential testing, which can improve prognostic accuracy in the CHR-P field (60).

**Figure 7:**
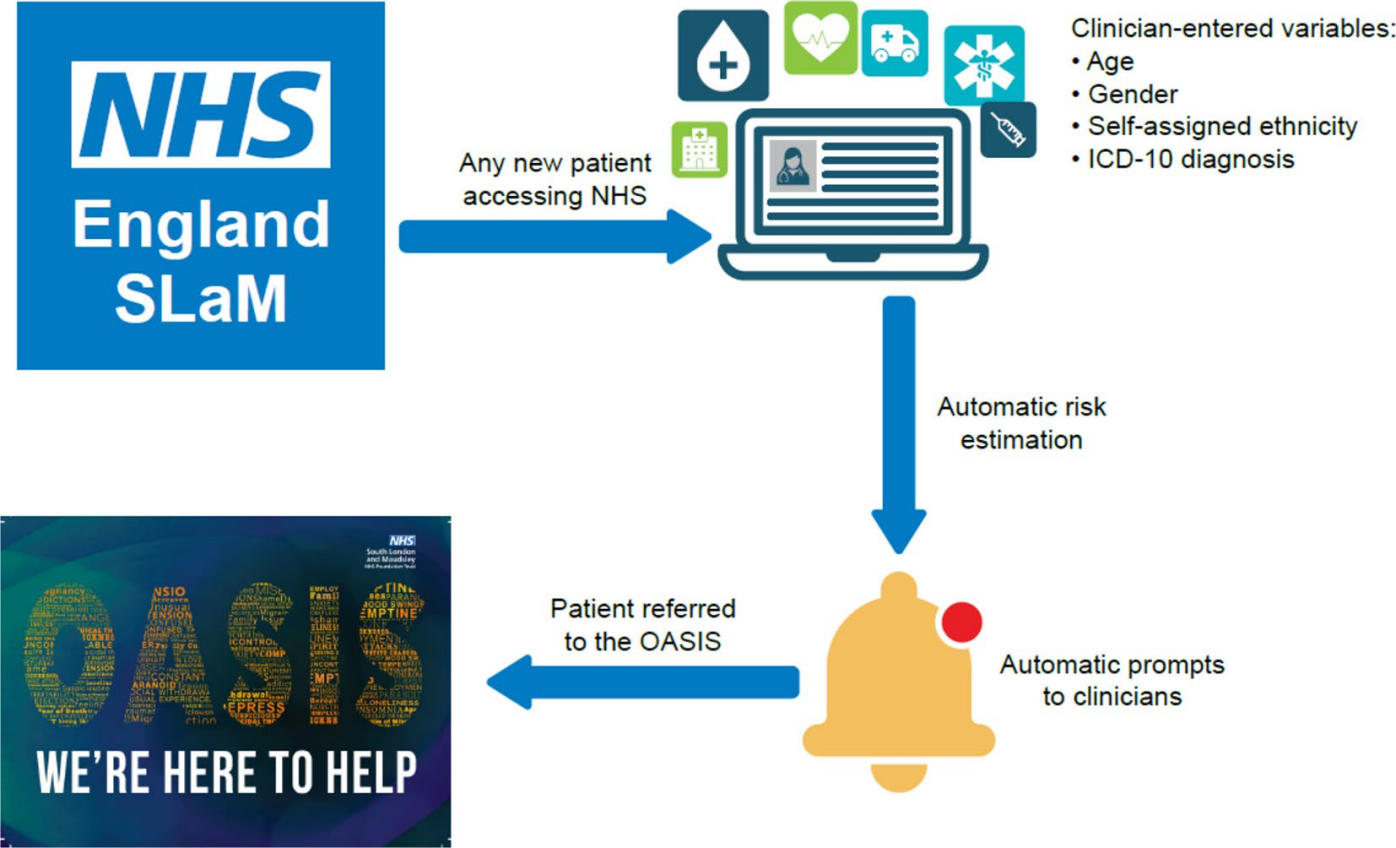
Potential clinical use of the individualized, clinically based, transdiagnostic risk calculator in secondary mental health care. For any new patient accessing the local NHS Trust (South London and Maudsley, UK), clinicians will enter the predictors on the electronic case register, as part of their clinical routine. The calculator, embedded in the local electronic health record, would then use the predictors to estimate the individual risk of developing psychosis over time. This information would then be shared with clinicians through automated alerts, inform their decision making, and promote appropriate referrals to the local early detection clinic (OASIS). From (55), Creative Commons Attribution License (CC BY).

This transdiagnostic risk calculator has been implemented in clinical care as part of an ongoing study funded by a Medical Research Council grant. Because external validation studies are rare, to our best knowledge, there are no other implementation studies of risk calculators for CHR-P patients. The proliferation of risk models in the CHR-P field as well as in psychiatry has occurred largely without appropriate attention to implementation challenges, resulting in many models that have little or no clinical impact (61). In fact, many more risk prediction models are published than are externally validated, and only a few of these are then implemented in the NHS (31). To achieve successful implementation, which is the true measure of a prediction model’s utility, we carefully considered potential implementation challenges from the beginning of the model building process. Because our aim was to improve the detection of individuals at risk of psychosis, it was necessary to screen a large NHS Trust at scale. To achieve this goal, we selected predictors that were already collected by clinicians as part of their clinical routine. Furthermore, the requirement of simple variables for implementation increases the number of datasets that could be used for the external validation of existing models, a current gap in the implementation of risk prediction models in psychiatry. The implementation study protocol for this transdiagnostic risk calculator has just been published (14). As indicated in **Figure 7**, this pilot study comprises of two subsequent phases: an *in vitro* phase of 1 month and an *in vivo* phase of 11 months.

The *in vitro* phase does not involve patients or clinicians, and it aims at developing and integrating the transdiagnostic risk calculator in the local electronic health register (primary outcome). The *in vivo* phase aims at addressing the clinicians’ adherence to the recommendations made by the transdiagnostic risk calculator (primary outcome) and other secondary feasibility parameters that are necessary to estimate the resources needed for its implementation. This pilot study is also the first to address the regulatory constraints that surround the automatic screening of electronic health-care records to detect patients at risk for psychosis [for a review, see (62)].

The study will be completed soon, and the results are expected over the next year. Should this study be successful, it will be followed by an effectiveness trial to test the real-world clinical and economic benefits of using this approach over standard care to detect patients at risk of psychosis in secondary mental health care. The complementary task would be to develop, validate, and implement risk calculators for the detection of patients at risk of psychosis in primary care, as highlighted in the following section.

### Improving The Detection Of Individuals At Risk In Primary Care

In the UK, most people with psychosis enter specialist secondary care via referral from their primary care physician (63), and there is some evidence that a shorter duration of untreated psychosis is associated with more primary care visits before diagnosis date (64). Primary care clinicians are therefore a vital part of the care pathway for people with psychosis, and it is consequently important that primary care clinicians can recognize a psychosis prodrome to expedite referral to specialist services for early treatment. Royal College of General Practitioners guidelines (65) stress the importance of detecting early signs and refer to some of the more common ones. There is evidence that the accuracy of psychosis diagnoses recorded on primary care electronic records is valid (66, 67), but there is also evidence that primary care physicians underidentify the more insidious symptoms (68). This is problematic because prodromal symptoms are frequently nonspecific and so may presage other health problems. In addition, most primary care physicians see very few new cases of psychosis per year and have little opportunity to increase personal experience in this area. There is also evidence (69) that there are barriers to referral for primary care when referring to specialist mental health services like CHR-P services. Therefore, there is a clear need for an accurate prognostic tool based in primary care. In line with the research program detailed above, it may be possible to use candidate predictors identified using clinical knowledge to develop and validate a prediction model based on primary care consultation data for nonpsychotic symptoms stored in electronic databases. Earlier studies (70) investigated the phases preceding psychosis, using a help-seeking general population sample from primary care consultation data collected before a diagnosis of psychosis and therefore unbiased by the presence of disorder. The sample used had a much larger number of cases (*n* = 11,690) than previous prospective studies. This method had the advantage of recording consultation events prospectively and should more accurately describe prodromal development. It was found that specific early behaviors and symptoms were strongly associated with a later diagnosis of psychosis, such as attention deficit hyperactivity-disorder-like problems, bizarre behavior, blunted affect, depressive-like problems, role functioning problems, social isolation, mania, obsessive–compulsive disorder-like problems, disordered personal hygiene, sleep disturbance, and suicidal behavior (including self-harm). The behaviors were cannabis use and cigarette smoking. The positive prognostic value of these behaviors and symptoms varied strongly with age and gender. There was also evidence of a pattern in consultation frequency per month for some of the prodromal behaviors and symptoms up to 5 years before diagnosis and evidence that people who are later diagnosed with psychosis are more frequent users of primary care services than those who do not develop psychosis. These findings can then be used to define candidate predictors for the development and validation of a psychosis detection and prediction model that can be used in primary care.

This research program is still ongoing, and the key methodological steps are summarized below. For the development and internal validation, we will conduct a population-based retrospective cohort study with a follow-up of ≥8 years. The Clinical Practice Research Datalink Gold (CPRD (71)) model will be used as a training dataset. CPRD Gold is a computerized database of anonymized longitudinal UK PC records, which covers approximately 22 million patients who are representative of the general UK population regarding age, sex, and ethnicity (72). Validation studies (73) report that the quality and completeness of data are high. To ensure that the recording of outcomes is complete, the CPRD Gold dataset will be linked to the Hospital Episode Statistics (HES) database (74), which records secondary health-care events in the UK. All patients within CPRD without a coded diagnosis of a psychotic disorder before 2010, but who consult for any mental health problem (a diagnosis or symptoms) from January 1, 2010 until the date of most recent general practitioner (GP) practice data download. Each patient will be regarded as at risk of a psychosis diagnosis from the date of the first consultation for a mental health problem of any nature. The end date will be the earliest date out of either the date on which HES records confirm a diagnosis of psychosis, or the date of data download, or the date the individual leaves the general practice or dies, or the practice ceases to provide data for CPRD.

The candidate predictors identified from our previous work (70) are described above. The primary outcome is any coded diagnosis of a psychotic disorder from HES records. We estimate that a CPRD dataset of the records of 300,000 people will contain at least 695 psychosis diagnoses, which exceeds the recommended event-per-variable ratio for risk prediction models (31). We will use robust multivariable and modern estimation methods employing shrinkage (75) (including LASSO) for variable selection, to guard against overfitting, along with a clinical judgement. Model performance will be assessed with calibration and discrimination, using well-established statistical performance measures (76). Time-varying predictors such as consultations per month will be incorporated within a Cox model. Internal model validation will quantify the model’s validity and the quality of predictors.

**Figure 8:**
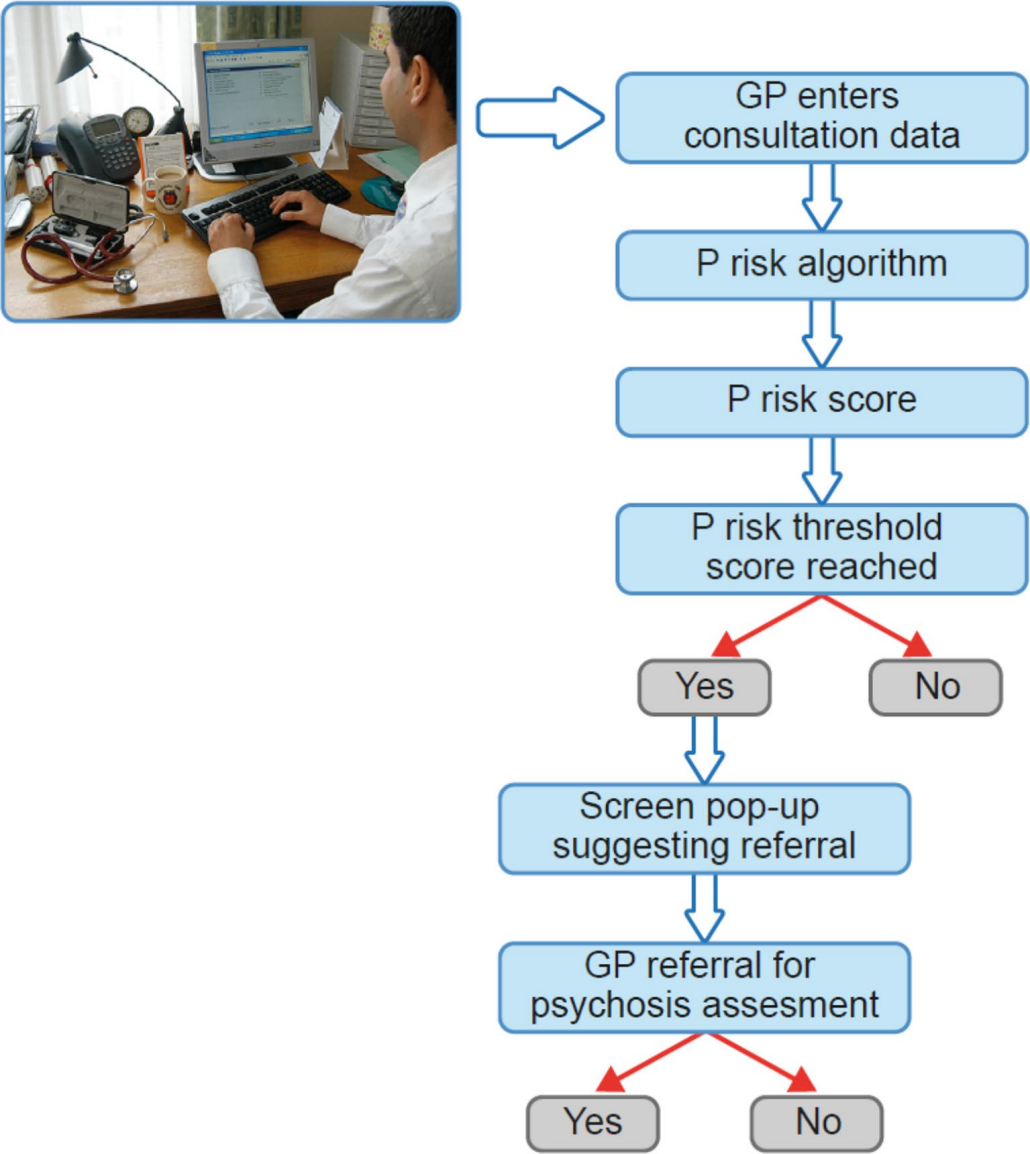
P-risk psychosis risk prediction algorithm operating on primary care data systems. New figure.

External validation will be conducted in the CPRD Aurum database linked to HES. GP practices included in CPRD Aurum only use EMIS primary care software for recording consultation data. Consequently, there is little or no overlap between the training and validation datasets. In internal model validation, calculations will be performed using bootstrap or cross-validation. In external validation, model performance measures will be calculated, and we will also report whether the prediction model is clinically useful using decision curve analysis to quantify the net benefit leading to an optimal decision threshold. Weighting of false versus true positive will be defined using clinician opinion (from the study team) and relevant literature (77). The final result will be a risk prediction algorithm—P risk (**Figure 8**).

Should this study be successful, it will lead to the next stage, which will be further external validation and pilot implementation of the P-risk algorithm in a live primary care setting. Following successful implementation, we would seek to test the effectiveness, cost effectiveness, and acceptability of P-risk using a randomized controlled trial design that would randomize a pop-up of the P-risk algorithm result to GPs and compare referral rates with GPs who do not receive the pop-up (see **Figure 8**).

### Improving The Detection Of Individuals At Risk In The Community

**Figure 9:**
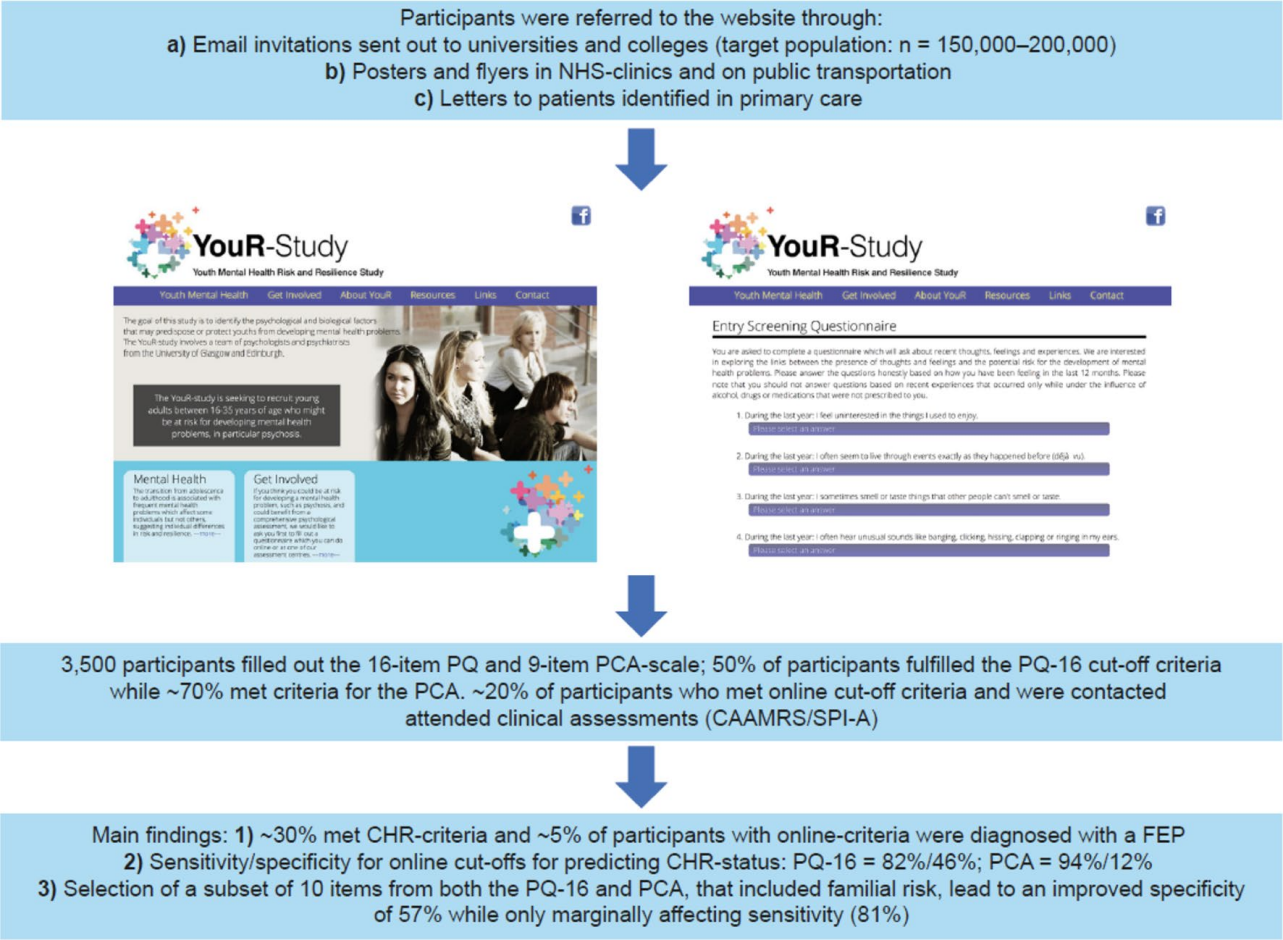
e-Health strategy to improve the detection of individuals at CHR-P in the community. CAARMS: Comprehensive Assessment of At-Risk Mental States; CHR-P: clinical high risk for psychosis; FEP: first episode psychosis; PCA: Questionnaire of Perceptual and Cognitive Aberrations; PQ-16: 16-item version of the Prodromal Questionnaire; SPI-A: Schizophrenia Proneness Instrument. New figure.

An obvious avenue for extending the detection of emerging psychosis to the community is through electronic mental health approaches. A recent study by Birnbaum et al. (78) surveyed the use of internet and social media resources among patients with FEP. The majority of patients actively sought information regarding mental health issues online and had positive attitudes toward online interventions. Accordingly, these data provide support for the idea that wider identification of psychosis may benefit from digital detection strategies (79). This possibility was tested as part of the Youth-Mental Risk and Resilience Study (80), a cross-sectional study to identify neurobiological mechanisms and predictors of psychosis risk. Specifically, the study implemented an online-screening tool (http://www.your-study.org.uk), which consists of a web-based questionnaire (81) that utilizes the 16-item version of the Prodromal Questionnaire (PQ-16) (82) and a 9-items of perceptual and cognitive aberrations for the assessment of basic symptoms. Such an approach is essential to minimize the caveats discussed above. While it is not recommended to directly screen the general population through CHR-P assessment tools, this can become viable if the samples have undergone some previous risk enrichment before. Using the PQ-16 ahead of the CHR-P assessment tool fulfills these requirements. In line with this approach, participants were invited to the study website via email invitations, posters, and flyers to take part in a study on mental health problems (81). It is estimated that a population of 150,000–200,000 students were contacted. Cut-off criteria for further clinical assessments were 6 or more positively endorsed items on the PQ-16 based on previous data, suggesting a correct classification of CHR-P criteria based on Comprehensive Assessment of At-Risk Mental States (CAARMS) interviews with high sensitivity and specificity (82). For the perceptual and cognitive aberrations, a cut-off score of 3 or more positively endorsed items was selected (**Figure 9**).

Three thousand five hundred participants completed the questionnaire online over a 4-year period. Our previous analysis (81) had shown that ∼50% participants fulfilled the PQ-16 cut-off criteria, while ∼70% met criteria for the perceptual and cognitive aberrations. Approximately 20% of participants who met online cut-off criteria and were contacted attended clinical assessments to establish CHR-P criteria based on the positive scale of the CAARMS (3) as well as through items of the Schizophrenia Proneness Instrument (adult version). Approximately one-third of participants who met online cut-off criteria and who were interviewed met CHR-P criteria. Importantly, a subset of individuals (∼5%) were also diagnosed with FEP and a substantial number of CHR-P participants had not received any intervention prior to the study. Receiver operating characteristic curve analysis revealed good to moderate sensitivity and specificity for predicting symptoms consistent with a CHR-P status based on online results for both CAARMS and Schizophrenia Proneness Instrument criteria (adult version) (sensitivity/specificity: PQ-16 = 82%/46%; perceptual and cognitive aberrations = 94%/12%) (81). To examine the possibility of improving the specificity of the online screening tool, we implemented a machine-learning approach that selected all 25 items from both the PQ-16 and the perceptual and cognitive aberrations in addition to demographical variables. Selection of a subset of 10 items from both PQ-16 and perceptual and cognitive aberrations that included familial risk lead to an improved specificity of 57% while only marginally affecting sensitivity (81%).

**Figure 10:**
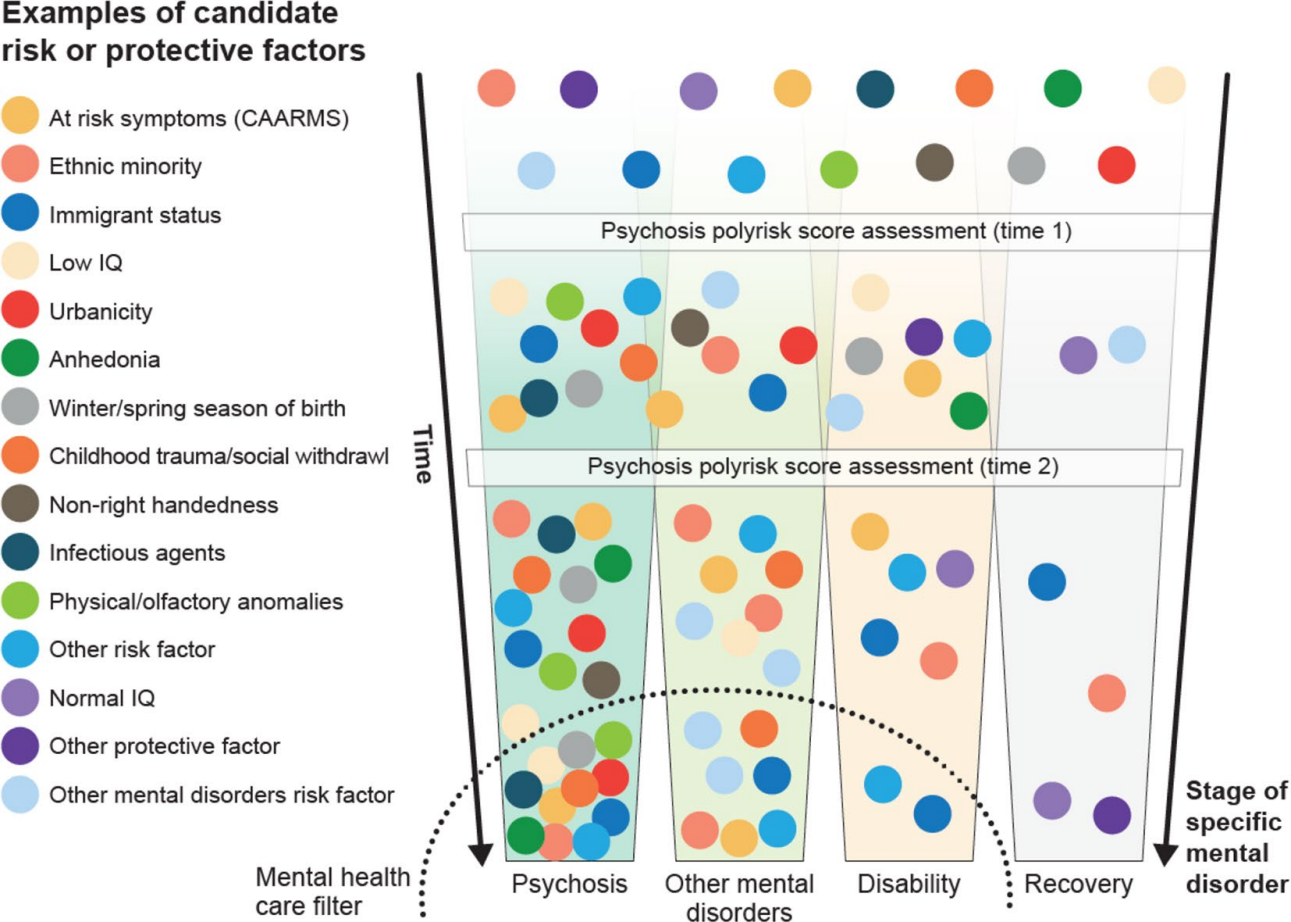
Putative Psychosis Polyrisk Score (PPS) assessment for the detection of at-risk individuals and the prediction of psychosis. Risk or protective factors that have been selected through umbrella reviews (38) are diluted during the preclinical stages but may accumulate as the individual progresses across different stages, until they trigger signs or symptoms and functional impairment that are associated with help-seeking behavior and access to mental health care. In the later stages, specific aggregations of risk and protective factors may be associated with specific clinical outcomes. Adapted from (36), Creative Commons Attribution License (CC BY).

These data provide the first evidence for the feasibility of using a digital detection tool to identify emerging psychosis in the community. However, several refinements are needed to improve this approach, in particular in regard to the specificity/sensitivity of the screener. This can be achieved, for example, by adding known risk factors for the development of psychotic disorders (21, 55) that can be efficiently integrated into a web- or app-based screening. Some members of our team are currently working on this line as part of a recently funded Wellcome Trust grant. Specifically, the online assessment will be complemented by the sequential use of the recently developed Psychosis Polyrisk Score (PPS, **Figure 10**). The use of the PPS can be particularly suited to detect those individuals who may be at risk of developing psychosis outside the CHR-P stage, as indicated above.

### Sequential Risk Assessment

The PPS leverages recent findings indicating that risk enrichment in CHR-P samples is accounted for by the accumulation of nongenetic factors such as parental and sociodemographic risk factors, perinatal risk factors, later risk factors, and antecedents (22). Examples of these risk factors are illustrated in **Figure 10**. The PPS additionally incorporates new meta-analytical evidence implicating specific risk factors that predict the onset of psychosis within CHR-P samples (83). The concurrent assessment of several demographic and environmental risk factors for psychosis may appear logistically unviable in clinical practice. However, it would be facilitated by a sequential testing procedure (60). For instance, all demographic and parental risk/protective factors, as well as some environmental (urbanicity, winter/spring season of birth) and later risk factors (adult life events, tobacco use, cannabis use, childhood trauma, traffic) can be self-administered or automatically extracted from electronic medical records or from geolocating applications that capitalize on recent e-health advancements (79). For the individuals whose predicted polyrisk of psychosis is over a certain threshold, a clinical comprehensive PPS assessment can be performed in a sequential fashion (60). Such an assessment may involve more accurate testing to collect the remaining risk factors: blood sampling to assess the exposure to infective agents and to estimate the polygenic risk, consulting obstetric records, or by interviewing the patients’ relatives and clinical interviews. Such an approach would additionally allow incorporating a dynamic assessment framework, which may better reflect the fluctuating course of the disorder. In line with these arguments, the e-detection tool that will be developed by this program could also incorporate behavioral data obtained through mobile phones, which could add important dimensions to the characterization of cognitive and behavioral deficits of participants at CHR-P. There is consistent evidence that cognitive functions, such as processing speed, are a core dysfunction of emerging psychosis (84), which could be assessed through digital phenotyping (85). In this context, there is also data evidence that speech analysis can be used to identify emerging psychosis that could be potentially an additional domain for a digital phenotyping approach (86, 87).

Digital detection of emerging psychosis in the community also faces several challenges; the most important is the significant prevalence of subthreshold psychotic experiences in the general population (49, 88). There is a significant phenomenological and clinical difference between subthreshold psychotic symptoms that are self-reported by youths in the general populations as opposed to the symptoms disclosed by youths who are accessing CHR-P services and undergoing a clinical interview (for details, see (8)). As noted above (33), these differences are likely to be associated with different level of pretest risk enrichment and, as such, with differential prognostic outcomes. Accordingly, future studies are needed to understand the ethical implications and establish the long-term outcomes of CHR-P populations recruited from the community through the use of prescreening e-health methods. Nonetheless, while these are important challenges to overcome, in the modern digital world, it is likely that e-health approaches such as the one presented here will have an increasing role to play in the future for the detection of emerging psychosis. This could be particularly true if these approaches are combined with complementary strategies targeting secondary and primary care.

## Conclusions

CHR-P instruments can provide reliable prognostic outcomes when they are employed in samples that have undergone risk enrichment during their recruitment. However, this enrichment substantially limits their detection power. Furthermore, there is evidence that psychosis onset may partially occur without a prior CHR-P stage and that nonpsychotic clinical risk states can precede FEP. To overcome these caveats, it is necessary to implement a clinical research program that integrates different but complementary detection approaches. A transdiagnostic individualized risk calculator could be used to automatically screen secondary mental health care to detect those at risk of psychosis and refer them to standard CHR-P assessment. Similar risk estimation tools for use in primary care are under development and promise to boost the detection of patients at risk in this setting. To improve the detection of young people who may be at risk of psychosis in the community, it is necessary to adopt e-health and sequential screening approaches that have been developed and are under refinement. These solutions are based on recent scientific evidence and can be potentially implemented into different contexts. Future research will test the cost effectiveness of these strategies, compared with current standards.

## Author Contributions

PF-P has conceived and led the study under the supervision of PU; PF-P, SS, JS, and PU wrote the initial draft of the manuscript, which was then collaboratively revised and approved by all authors.

## Funding

Editorial support in the form of graphical improvement of the figures and copy editing was provided by Sam Halliwell, PhD, of Fishawack Communications Ltd, and was funded by Boehringer Ingelheim International GmbH.

## Conflict of Interest

PF-P has received honoraria, advisory fees, or research funds from Lundbeck LTD, Menarini, Boehringer Ingelheim International GmbH outside the current study. The remaining authors declare that the research was conducted in the absence of any commercial or financial relationships that could be construed as a potential conflict of interest.
